# Metabolomics and Precision Medicine in Resistant Hypertension: Pathophysiological Insights, Biomarker Discovery, and Translational Strategies

**DOI:** 10.1155/ijhy/9656975

**Published:** 2026-07-14

**Authors:** Yu Ra Lee, Miri Park, Yoon Shin Cho, Ho-Young Park

**Affiliations:** ^1^ Food Functionality Research Division, Korea Food Research Institute, Wanju-gun, Jeollabuk-do, 55365, South Korea, kfri.re.kr; ^2^ Department of Biomedical Science, Hallym University, Chuncheon-si, Gangwon-do, 24252, South Korea, hallym.ac.kr; ^3^ Department of Food Biotechnology, Korea National University of Science and Technology, Daejeon, 34113, South Korea

**Keywords:** biomarker discovery, metabolomics, multiomics, precision medicine, resistant hypertension

## Abstract

Resistant hypertension (RH), defined as uncontrolled blood pressure despite the use of at least three optimally dosed antihypertensive agents, including a diuretic, remains a major clinical challenge associated with elevated cardiovascular risk. Metabolomics offers a dynamic approach to characterize biochemical perturbations related to amino acid metabolism, lipid remodeling, mitochondrial dysfunction, oxidative stress, renal impairment, and gut microbiota–derived metabolites. However, current evidence remains limited by small sample sizes, cross‐sectional designs, heterogeneous definitions of RH, inadequate exclusion of pseudoresistance, medication confounding, and limited external validation. This structured narrative review synthesizes RH‐specific metabolomic evidence and distinguishes it from findings extrapolated from broader hypertension populations. We further discuss methodological challenges, replication gaps, pharmacometabolomic confounding, and validation standards required for clinical implementation. Integrating metabolomics with clinical phenotyping, genomics, proteomics, and microbiome profiling may eventually support RH phenotyping, treatment–response prediction, and biomarker‐guided precision medicine, but large longitudinal cohorts with confirmed true RH are needed before clinical translation.

## 1. Introduction

Resistant hypertension (RH) is defined as blood pressure that remains above target despite concurrent use of three antihypertensive agents of different classes, including a diuretic, at optimal or maximally tolerated doses [1]. Patients requiring four or more drugs to achieve control are also classified as having RH. This clinical entity was formally recognized in the 2008 American Heart Association statement and subsequently updated in European and other international guidelines, underscoring its importance in cardiovascular risk management [2]. Distinguishing true RH from pseudoresistance due to poor adherence, white‐coat effect, or inadequate therapy is critical, as misclassification can lead to inappropriate interventions [3].

Globally, RH affects 10%–20% of hypertensive patients, with higher prevalence in those with chronic kidney disease (CKD), diabetes, and obesity and in older adults [4]. However, prevalence estimates vary depending on diagnostic criteria, clinical settings, and regional factors, with limited data from developing countries [5]. Given the increasing burden of metabolic disease and aging populations, RH prevalence is expected to rise, posing significant challenges for healthcare systems.

Clinically, RH is associated with increased risk of myocardial infarction, stroke, heart failure, left ventricular hypertrophy, and progressive renal dysfunction, thereby driving healthcare costs and utilization [6]. Despite the availability of numerous pharmacologic and nonpharmacologic interventions, RH management often relies on polypharmacy, which increases the risk of side effects, poor adherence, and reduced quality of life [7]. Device‐based interventions such as renal sympathetic denervation have produced inconsistent results in randomized controlled trials, and individualized treatment strategies remain lacking [8].

A major barrier to effective RH management is the absence of reliable biomarkers to differentiate true from pseudoresistance, stratify patients by pathophysiological phenotype, and predict therapeutic responsiveness [3]. This underscores the need for deeper mechanistic understanding and novel frameworks for diagnosis and therapy.

Metabolomics, the comprehensive study of small‐molecule metabolites in biological systems, has emerged as a powerful tool to investigate complex diseases like RH [9]. Unlike genomics and transcriptomics, which capture static or upstream molecular information, metabolomics reflects the dynamic biochemical phenotype influenced by genetic, environmental, dietary, and lifestyle factors. It captures the cumulative output of multiple regulatory networks and is especially suitable for exploring multifactorial diseases [10]. In hypertension research, metabolomics enables the identification of metabolic signatures linked to vascular dysfunction, neurohormonal activation, inflammation, oxidative stress, and renal impairment [11].

Available metabolomic studies in RH and related hypertensive phenotypes suggest perturbations in key metabolic pathways such as amino acid metabolism, lipid metabolism, the tricarboxylic acid (TCA) cycle, and oxidative stress–related metabolites [12]. These findings not only provide mechanistic insight into the underlying pathophysiology but also offer potential biomarkers for diagnosis, stratification, and therapeutic monitoring. Additionally, metabolomics can be integrated with other omics platforms, including genomics, proteomics, and microbiome data, to provide a systems‐level understanding of RH [13]. Despite challenges related to analytical variability, data interpretation, and clinical translation, metabolomics holds considerable potential for advancing precision medicine in RH.

This review aims to synthesize current knowledge on the pathophysiology, epidemiology, and clinical implications of RH, highlight key findings from recent metabolomic studies, and discuss emerging opportunities for biomarker discovery and personalized management. Through a systems biology lens, we explore how metabolomics could reshape the clinical approach to RH and support more effective, individualized interventions.

The novelty of this review lies in three aspects. First, we distinguish metabolomic findings directly derived from RH cohorts from those extrapolated from essential hypertension, prehypertension, or adjacent vascular phenotypes. Second, we critically evaluate major sources of bias in RH metabolomics, including pseudoresistance, medication exposure, comorbid CKD, diabetes, obesity, and lack of longitudinal validation. Third, we propose a translational roadmap in which metabolomics is positioned not as a standalone diagnostic tool but as an adjunctive approach for RH phenotyping, treatment–response prediction, adherence assessment, and biomarker‐guided precision medicine.

## 2. Methods: Literature Search and Study Selection

This article was designed as a structured narrative review rather than a systematic review. We searched PubMed/MEDLINE, Web of Science, and Scopus for English‐language articles published from database inception to May 21, 2026. The final search was conducted on May 21, 2026. Search terms included combinations of the following terms: “resistant hypertension,” “apparent treatment‐resistant hypertension,” “true resistant hypertension,” “metabolomics,” “metabolomic profiling,” “biomarker,” “multi‐omics,” “pharmacometabolomics,” “ambulatory blood pressure monitoring,” and “medication adherence.” Studies were prioritized if they included human participants with RH, metabolomic profiling, treatment–response assessment, or multiomics integration. Studies of essential hypertension, prehypertension, pulmonary arterial hypertension, or general cardiovascular/metabolic disease were included only when they provided mechanistic context or comparator evidence and were clearly distinguished from RH‐specific evidence.

## 3. Definition, Epidemiology, and Diagnostic Confirmation of RH

### 3.1. Epidemiology and Risk Factors of RH

RH is increasingly recognized as a global public health concern due to its growing prevalence and strong association with cardiovascular and renal morbidity [14]. Epidemiological estimates suggest that RH affects approximately 10%–20% of the hypertensive population, though reported prevalence rates vary depending on the criteria used to define RH, the inclusion of pseudoresistant cases, and the population under study [15]. In clinical trial populations and tertiary care settings, prevalence may be overestimated due to referral bias, while in primary care, RH may be underdiagnosed due to limited confirmatory diagnostic testing such as ambulatory blood pressure monitoring (ABPM) [16].

In the United States, data from the National Health and Nutrition Examination Survey have shown that nearly 15%–20% of treated hypertensive patients fulfill the criteria for apparent RH, although a significant portion of these may have pseudoresistance due to poor adherence or suboptimal therapeutic regimens [17]. In contrast, European studies report a prevalence of 10%–15% in community‐based cohorts [18]. In Asia, including Korea and Japan, data remain limited but suggest rising trends correlating with increasing obesity, metabolic syndrome, and aging populations [19]. Differences in lifestyle, dietary sodium intake, healthcare access, and medication patterns likely contribute to regional disparities [20].

Several demographic and clinical risk factors have been consistently associated with RH [21]. Advancing age is one of the most robust predictors, as vascular stiffness and arterial remodeling become more pronounced in older adults. Black individuals are disproportionately affected by RH, exhibiting higher prevalence and greater end‐organ damage, potentially due to enhanced sodium sensitivity and more pronounced renin–angiotensin–aldosterone system (RAAS)–independent mechanisms [22]. Female sex has also been implicated as a risk factor in some studies, though findings are mixed.

Comorbid conditions strongly predispose individuals to RH [23]. CKD is particularly relevant, as impaired sodium excretion and fluid overload exacerbate blood pressure elevation and treatment resistance. Diabetes mellitus, especially when accompanied by insulin resistance, contributes through vascular dysfunction, sympathetic overactivity, and inflammation [24]. Obesity plays a central role, not only as a risk factor for hypertension onset but also as a contributor to RH via adipokine imbalance, neurohormonal activation, and structural changes in the kidney and vasculature [25].

Lifestyle and behavioral factors are equally important. High dietary sodium intake is a well‐established contributor to volume‐dependent hypertension and may blunt the effect of antihypertensive medications [26]. Excessive alcohol consumption, physical inactivity, and poor sleep hygiene, including obstructive sleep apnea, further increase RH risk [27]. Medications such as nonsteroidal anti‐inflammatory drugs, corticosteroids, oral contraceptives, and sympathomimetic agents may also interfere with blood pressure control and should be carefully reviewed in RH cases [28].

Importantly, RH is often accompanied by a clustering of adverse cardiometabolic traits, including left ventricular hypertrophy, arterial stiffness, microalbuminuria, hyperuricemia, and metabolic syndrome [29]. These overlapping pathologies complicate diagnosis and management while elevating long‐term risks for cardiovascular events. Accordingly, early identification of high‐risk individuals using risk stratification tools and comprehensive evaluation is critical to prevent progression and optimize treatment outcomes.

### 3.2. Diagnostic Confirmation of True RH and Implications for Metabolomics

A critical methodological issue in RH metabolomics is the accurate confirmation of true RH. Apparent RH may result from poor medication adherence, white‐coat effect, inadequate drug dosing, suboptimal diuretic use, or interfering medications. Therefore, metabolomic studies that do not confirm adherence or out‐of‐office blood pressure may inadvertently capture metabolic signatures of pseudoresistance rather than true treatment resistance. Ideally, RH metabolomics cohorts should document optimized antihypertensive therapy, including a diuretic; exclude white‐coat hypertension using ambulatory or home blood pressure monitoring; assess medication adherence using pharmacy refill data, pill counts, electronic monitoring, or drug/metabolite assays; and evaluate secondary causes of hypertension. These diagnostic safeguards are essential for distinguishing biological treatment resistance from behavioral, measurement‐related, or therapeutic causes of uncontrolled blood pressure.

## 4. Pathophysiology of RH

The pathophysiology of RH is multifactorial and involves a complex interplay of neurohormonal dysregulation, renal impairment, vascular remodeling, inflammation, oxidative stress, and metabolic dysfunction [30]. These interacting systems contribute to persistent elevation of blood pressure despite the use of multiple antihypertensive agents [31].

One of the central mechanisms implicated in RH is the overactivation of the RAAS [32]. Excessive aldosterone promotes sodium retention, extracellular fluid expansion, and increased vascular resistance [33]. In RH, aldosterone‐mediated effects extend beyond volume overload to include vascular fibrosis, endothelial dysfunction, and heightened sympathetic nervous system (SNS) activity [34]. These changes lead to impaired vasodilation, arterial stiffness, and abnormal baroreflex sensitivity [35].

Chronic activation of the SNS is another hallmark of RH pathogenesis [36]. Elevated sympathetic outflow results in increased cardiac output, vasoconstriction, and renal sodium reabsorption. Additionally, it can reduce responsiveness to antihypertensive therapies and potentiate insulin resistance, further exacerbating blood pressure dysregulation [37].

Renal dysfunction is both a cause and consequence of RH [38]. Reduced glomerular filtration rate and impaired natriuresis contribute to volume expansion, while renal ischemia perpetuates RAAS activation and sympathetic drive [39]. Furthermore, structural abnormalities in renal vasculature and tubular damage hinder therapeutic response [40]. Patients with coexisting CKD often exhibit a more severe and refractory hypertensive phenotype [41].

Inflammatory processes play a significant role in RH pathophysiology [42]. Elevated circulating levels of inflammatory cytokines such as interleukin‐6, tumor necrosis factor‐alpha, and C‐reactive protein have been documented in RH patients [43]. These proinflammatory mediators impair endothelial function, promote oxidative stress, and amplify RAAS activity, contributing to vascular dysfunction and remodeling [44].

Oxidative stress, characterized by an imbalance between reactive oxygen species (ROS) production and antioxidant defense mechanisms, further aggravates endothelial damage and nitric oxide (NO) deficiency [45]. Decreased NO bioavailability impairs vasodilation and promotes vascular stiffness, both of which are key features of RH. Mitochondrial dysfunction, which underlies increased ROS generation and defective energy metabolism, has also been implicated [46].

Recent research suggests that gut microbiota may influence blood pressure regulation through the production of metabolites such as short‐chain fatty acids (SCFAs) and trimethylamine N‐oxide (TMAO) [47]. These microbial‐derived compounds can modulate inflammation, oxidative stress, and RAAS activity, potentially contributing to RH development [48]. Dysbiosis may thus represent a novel pathophysiological axis in RH.

Metabolic abnormalities, including insulin resistance, altered lipid profiles, and impaired glucose tolerance, are frequently observed in RH and are linked to the broader cardiometabolic syndrome. These alterations can directly impair vascular homeostasis and exacerbate sympathetic and RAAS activation [49].

Altogether, the pathophysiology of RH reflects a convergence of hemodynamic, hormonal, inflammatory, and metabolic disturbances. A systems‐level understanding of these mechanisms is essential for identifying novel therapeutic targets and for advancing the use of omics‐based approaches, such as metabolomics, in RH research and management.

## 5. Therapeutic Limitations and Unmet Clinical Needs in RH

Despite broad therapeutic options, management of RH remains difficult. Standard care typically escalates to stepwise combination therapy with a diuretic, calcium‐channel blocker, renin–angiotensin system inhibitor, and often a mineralocorticoid receptor antagonist [50]. However, this polypharmacy approach is associated with increased risks of adverse drug reactions, poor medication adherence, and diminished quality of life, particularly in elderly patients or those with multiple comorbidities.

Clinical inertia and limited awareness of RH diagnostic criteria further contribute to underrecognition and suboptimal treatment. Many patients are not evaluated with ABPM to distinguish true RH from pseudoresistance [28]. Moreover, the lack of validated biomarkers hampers early diagnosis and patient stratification, making it difficult to identify individuals who would benefit most from intensive or targeted interventions.

Device‐based therapies, such as renal sympathetic denervation and baroreceptor activation therapy, have generated interest as nonpharmacological options for RH. While early studies showed promise, large randomized controlled trials such as SYMPLICITY HTN‐3 failed to demonstrate consistent efficacy, possibly due to heterogeneous study populations and inadequate patient selection criteria [51]. Similarly, newer agents targeting aldosterone biosynthesis or sodium transport mechanisms are under investigation but remain experimental.

Nonpharmacological strategies, including dietary modification (e.g., sodium restriction and DASH diet), weight loss, increased physical activity, and reduction of alcohol intake, are foundational to RH management [52]. However, adherence to these lifestyle interventions is often poor in real‐world settings, limiting their long‐term effectiveness. Cognitive‐behavioral strategies, mobile health technologies, and team‐based care models may help address this gap but require more widespread implementation [53].

Critically, current care pathways largely follow an empirical, “one‐size‐fits‐all” escalation model that neglects biological heterogeneity in RAAS activity, sodium handling, sympathetic tone, vascular reactivity, and metabolic state. Precision frameworks that incorporate molecular and physiological profiling are therefore needed to guide individualized therapy and improve outcomes [54].

In summary, the management of RH is hindered by limitations in diagnostic precision, therapeutic personalization, and long‐term adherence strategies. There is a pressing need to develop and validate novel biomarkers and risk prediction models, implement comprehensive care pathways, and leverage systems biology, including metabolomics, to guide individualized treatment strategies that can improve clinical outcomes.

## 6. Role of Metabolomics in Understanding and Managing RH

**FIGURE 1:**
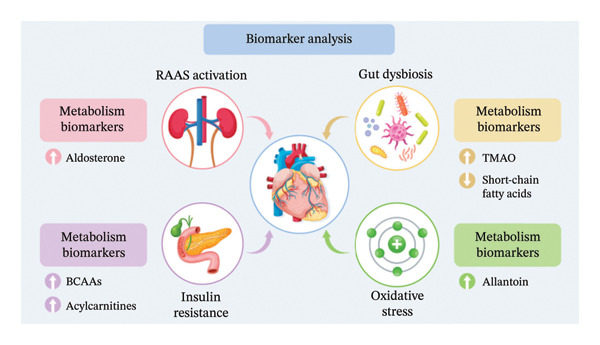
Pathophysiological mechanisms in resistant hypertension: Where metabolomics fits. This schematic diagram summarizes the key biological pathways implicated in resistant hypertension, including neurohormonal activation, vascular dysfunction, inflammation, oxidative stress, and metabolic disturbances. Metabolomics offers insight into these processes by identifying altered metabolites across lipid, amino acid, and energy metabolism, supporting its role in biomarker discovery and personalized therapeutic strategies.

Metabolomics, the comprehensive profiling of small‐molecule metabolites in biological specimens such as blood, urine, or tissue, is a rapidly growing field with significant relevance for complex disorders like RH. Unlike genomics or transcriptomics, which offer a blueprint of biological potential, metabolomics provides a dynamic and real‐time snapshot of physiological states shaped by gene‐environment interactions [55], lifestyle factors, comorbidities, and therapeutic interventions. The integration of metabolomics into RH research provides an opportunity to bridge molecular alterations with clinical phenotypes. Figure 1 illustrates the key pathophysiological mechanisms involved in RH and highlights where metabolomics can provide mechanistic and diagnostic insights. As a result, it captures the actual phenotype of disease processes more effectively and offers an integrative view of metabolic dysfunctions in RH.

In RH, metabolomics has demonstrated its utility in uncovering pathophysiologic mechanisms, identifying candidate biomarkers, and exploring treatment response variability [13]. Recent studies employing mass spectrometry and nuclear magnetic resonance (NMR)–based platforms have revealed consistent perturbations in metabolic pathways, including:

Amino acid metabolism: Elevations in branched‐chain amino acids (BCAAs), glutamate, and homocysteine, which may be linked to vascular inflammation, endothelial dysfunction, and insulin resistance [56].

Lipid metabolism: Changes in lysophosphatidylcholines (LysoPCs), sphingolipids, and acylcarnitines, suggesting impaired fatty acid oxidation and mitochondrial dysfunction [11].

Energy metabolism: Disruptions in the TCA cycle intermediates and glycolytic metabolites, reflecting altered bioenergetic status in RH patients [57].

Oxidative stress markers: Increased levels of metabolites such as allantoin or malondialdehyde indicate heightened oxidative stress and redox imbalance [58].

These findings may have potential clinical relevance, although their RH specificity and clinical utility remain to be validated as many of the identified metabolites correlate with blood pressure levels [59], arterial stiffness [60], renal function [61], and target organ damage [62]. Importantly, if validated in independent cohorts, metabolic signatures may eventually help stratify RH patients into subgroups with differential responsiveness to mineralocorticoid receptor antagonists, renin inhibitors, or dietary interventions.

Beyond diagnostic utility, metabolomics offers translational applications in drug development, therapeutic monitoring, and reverse phenotyping. For instance, monitoring metabolomic shifts before and after antihypertensive therapy may help distinguish responders from nonresponders and optimize treatment regimens [63]. Furthermore, integrating metabolomics with pharmacogenomics can uncover pharmacometabolomic profiles predictive of drug efficacy or adverse events [64].

Emerging research is also exploring the role of gut microbiota–derived metabolites, such as SCFAs, TMAO, and bile acids, in RH pathogenesis [65]. These molecules influence vascular tone, inflammation, and sodium handling and serve as promising targets for microbiome‐modulating therapies [66].

Despite its promise, several limitations must be addressed to advance metabolomics in RH research: interplatform variability, cohort heterogeneity, lack of longitudinal data, and limited standardization across studies [67]. To illustrate the current state of research, Table 1 summarizes metabolomics studies relevant to RH, including RH‐specific cohorts, treatment–response studies, general hypertension cohorts, prehypertension, incident hypertension, and adjacent hypertensive phenotypes [12, 57, 63, 68–82]. These studies highlight altered metabolic pathways, candidate biomarkers, and methodological diversity across study designs, but they also emphasize that most metabolomic findings in hypertension are not yet specific to confirmed RH. While these findings offer valuable insights, differences in analytical platforms, sample sizes, and population characteristics continue to limit reproducibility and generalizability. Therefore, despite encouraging preliminary data, substantial methodological refinements are necessary to establish the clinical utility of metabolomics in RH. Moving forward, large‐scale, multicenter, longitudinal metabolomics studies with harmonized methodologies and integration with other omics layers (genomics, proteomics, and microbiome) will be critical [13]. Because only a limited number of metabolomics studies have directly examined patients with confirmed RH, findings from essential hypertension, prehypertension, pulmonary arterial hypertension, or other cardiometabolic conditions should not be interpreted as RH‐specific biomarkers. Instead, these studies provide mechanistic context for pathways that may be relevant to RH, including amino acid metabolism, lipid remodeling, mitochondrial dysfunction, oxidative stress, renal impairment, and gut microbiota–derived metabolites. Throughout this review, we therefore distinguish RH‐specific findings from extrapolated evidence and hypothesis‐generating observations.

**TABLE 1:** Evidence map of metabolomics studies relevant to resistant hypertension: RH‐specific, general hypertension, and adjacent hypertensive phenotypes.

No.	Profile type	Sample	Patient population/comparison	Evidence category	RH confirmation	Key pathways or metabolites	Interpretation for RH	Reference
1	Untargeted	Plasma	Resistant hypertension vs. controlled hypertension	RH‐specific	RH diagnosis reported; ABPM/HBPM and adherence confirmation should be checked in the original study	Amino acids, organic acids, lipids, indoles; ↑ ornithine, phenylalanine, uric acid, phenylpropionic acid, undecanedioic acid, 5‐hydroxyindoleacetic acid; ↓ octylamine, oxononanoic acid, hydroxyeicosatetraenoic acid, hydroxydocosahexaenoic acid	RH‐specific candidate findings, but exploratory and not clinically validated	[12]
2	Untargeted	Urine	Resistant hypertension; metabolic prediction of treatment response	RH‐specific/treatment–response study	RH diagnosis reported; treatment–response assessment included; ABPM/HBPM and adherence confirmation should be checked in the original study	Citric acid metabolism, TCA cycle intermediates, oxidative stress–related pathways; citrate and oxaloacetate‐related alterations	RH‐relevant exploratory markers and potential treatment–response candidates; external validation required	[57]
3	Untargeted	Plasma	Essential hypertension patients before and after antihypertensive drug administration	General hypertension/pharmacometabolomics	Not applicable	Drug‐related changes in acylcarnitines, long‐chain fatty acids, uric acid, steroid sulfates, inorganic sulfates, phosphate, and fatty acid oxidation‐related metabolites	Useful for understanding medication‐related metabolomic confounding; not RH‐specific	[63]
4	Untargeted	Plasma	Hypertensive patients with early vascular aging vs. hypertensive patients without early vascular aging	Adjacent hypertensive phenotype	Not applicable	Phospholipid metabolism; ↓ lysophosphatidylcholines including LPC 18:2, LPC 16:0, LPC 18:0, and LPC 18:1	Provides vascular aging and lipid metabolism context; not RH‐specific	[68]
5	Untargeted	Plasma	Hypertension vs. healthy control	General hypertension	Not applicable	Lipid metabolism; ↓ free cholesterol and ether phospholipids, including phosphatidylcholine‐O and phosphatidylethanolamine‐O species	General hypertension lipidomic evidence; mechanistic context only	[69]
6	Targeted	Plasma	Hypertension stages/blood pressure progression vs. normotensive controls	General hypertension/BP progression	Not applicable	Fatty acids and glycerolipids; ceramides, triacylglycerols, diacylglycerols, monoacylglycerols, oleic acid, and cholesteryl esters	Supports metabolic risk and BP progression biology; extrapolated evidence for RH	[70]
7	Targeted	Plasma	Men and women with arterial hypertension vs. healthy controls	General hypertension/sex‐specific phenotype	Not applicable	Sphingomyelins, phosphatidylcholines, hydroxyproline, symmetric dimethylarginine, total dimethylarginine, histidine, spermidine, spermine, and LysoPC species	Highlights sex‐related metabolic differences in hypertension; not RH‐specific	[71]
8	Targeted	Plasma	Prehypertension vs. healthy control	Prehypertension	Not applicable	Fatty acids and LysoPCs; ↑ myristic acid, palmitoleic acid, linoleic acid, oleic acid, stearic acid, eicosapentaenoic acid, arachidonic acid, and LysoPCs	Early blood pressure/metabolic risk context; not RH‐specific	[72]
9	Targeted	Plasma	Hypertension vs. healthy control	General hypertension	Not applicable	Fatty acid, glucose, amino acid, purine, and tryptophan metabolism; altered citrulline, glycine, oleic acid, uric acid, sugars, tyrosine, branched‐chain amino acids, tryptophan, and α‐tocopherol	General hypertension candidate metabolites; extrapolated evidence only	[73]
10	Targeted	Serum	Essential hypertension vs. healthy control	General hypertension	Not applicable	Amino acid and fatty acid oxidation pathways; ↑ ornithine/citrulline ratio; ↓ glycine and acylcarnitines	General hypertension metabolic signature; not RH‐specific	[74]
11	Targeted	Serum	Essential hypertension vs. healthy control	General hypertension	Not applicable	Fatty acids, glucose‐related metabolites, and urea cycle metabolites; altered monoacylglycerols, oleic acid, sugars, myo‐inositol, aspartic acid, glutamine, and ornithine	Provides general metabolic context for hypertension; extrapolated evidence	[75]
12	Targeted	Serum	Endocrine hypertension vs. primary hypertension	Adjacent hypertensive phenotype	Not applicable	Acylcarnitines, fatty acids, glycerophospholipids, bile secretion‐related metabolites, amino acids, pyruvate metabolism, and TCA cycle metabolites	Mechanistic context only; not direct RH evidence	[76]
13	Targeted	Serum	H‐type hypertension vs. healthy control	General hypertension/specific hypertension subtype	Not applicable	Fatty acids, amino acids, and organic acids; altered palmitic acid, stearic acid, linoleic acid, alanine, histidine, isoleucine, leucine, lysine, methionine, ornithine, proline, taurine, threonine, cis‐aconitic acid, creatinine, malic acid, oxalic acid, and pyruvic acid	Relevant to metabolic heterogeneity in hypertension; not RH‐specific	[77]
14	Targeted	Plasma	Black hypertensive patients vs. White hypertensive patients	General hypertension/population‐specific phenotype	Not applicable	Amino acids including alanine, glycine, serine, methionine, dimethylglycine, glutamine, 4‐hydroxyproline, and histidine	Highlights population‐related metabolic variation in hypertension; extrapolated evidence	[78]
15	Targeted	Plasma	African American and White American individuals with high blood pressure vs. controls	General hypertension/population‐specific phenotype	Not applicable	Amino acid and microbiota‐related metabolites; leucine, quinolinic acid, sulfoacetaldehyde, betaine, phenylalanine, 5‐aminolevulinic acid, anserine, 4‐oxoproline, and α‐hydroxyisobutyric acid	Supports population and microbiome‐related metabolic heterogeneity; not RH‐specific	[79]
16	Targeted	Serum	Incident hypertension risk in a nested case–control study	General hypertension/incident hypertension	Not applicable	Amino acid–related metabolites; phenylalanine, tyrosine, shikimic acid, glycine, and threonine	Risk‐prediction context for hypertension; extrapolated evidence	[80]
17	Targeted	Plasma	Young hypertensive men vs. healthy controls	General hypertension	Not applicable	Organic acids, amino acids, carbohydrates, lipids, and purine‐related metabolites; oxalic acid, fumaric acid, branched‐chain amino acids, glycine, methionine, ornithine, glutamine, citrulline, lysine, tyrosine, tryptophan, glycerol, adenine, capric acid, pyrophosphate, and uric acid	General hypertension metabolic network evidence; not RH‐specific	[81]
18	Targeted	Serum	Incident hypertension among Black participants	General hypertension/incident hypertension	Not applicable	Benzoate‐related microbial fermentation product and sex steroid hormone metabolites; 4‐hydroxyhippurate, androsterone sulfate, epiandrosterone sulfate, and related steroid sulfates	Microbiome‐ and steroid‐related risk context; hypothesis‐generating only	[82]

*Note:* Evidence categories were assigned to distinguish studies directly conducted in RH cohorts from those performed in broader hypertension, prehypertension, incident hypertension, treatment–response, or adjacent vascular/metabolic phenotypes. Studies not conducted in RH populations were included only to provide mechanistic or comparator context and should not be interpreted as evidence of RH‐specific biomarkers. “RH confirmation” indicates whether the study population was directly relevant to RH and whether confirmation of true RH using out‐of‐office BP measurement, medication adherence assessment, and optimized treatment was reported or should be verified in the original article. LPC, lysophosphatidylcholine; LysoPC, lysophosphatidylcholine; TCA, tricarboxylic acid.

Abbreviations: ABPM, ambulatory blood pressure monitoring; BP, blood pressure; HBPM, home blood pressure monitoring; RH, resistant hypertension.

Metabolomics offers a systems medicine lens for RH, linking perturbed metabolic pathways to vascular, renal, neurohormonal, and inflammatory phenotypes; enabling biomarker‐guided precision care; and providing pharmacodynamic readouts to individualize therapy. Its clinical impact will hinge on standardized workflows, cross‐cohort validation, and seamless fusion with genomic, proteomic, and microbiome data.

## 7. Current Metabolomics Evidence and Interpretation in RH

### 7.1. Summary of Key Metabolomics Studies in RH

Recent technological advances have facilitated in‐depth metabolomic analyses of RH, uncovering novel biochemical alterations and laying the foundation for biomarker discovery and mechanistic research [83]. Several studies employing various analytical platforms, such as liquid chromatography–mass spectrometry (LC–MS), gas chromatography–mass spectrometry, and NMR, have identified consistent metabolic disruptions in RH patients across different biospecimens, including plasma, urine, and tissue [84].

For example, Martin‐Lorenzo et al. performed an untargeted LC–MS‐based metabolomic study on urine samples from RH patients and found elevated levels of citrate and oxaloacetate. These changes were associated with oxidative stress, inflammation, and endothelial dysfunction, key features of RH pathogenesis. Furthermore, metabolomic signatures distinguished RH from controlled hypertension, indicating diagnostic potential [57].

Additionally, several studies have implicated gut microbiota–derived metabolites in RH. Elevated plasma levels of TMAO, a microbial metabolite linked to vascular inflammation and renal dysfunction, were reported in patients with uncontrolled hypertension and RH. This finding underscores the role of gut microbiome–host interactions in RH pathophysiology [85].

Despite these promising insights, several limitations constrain current metabolomics studies in RH. Sample sizes remain small, study populations are often heterogeneous, and few investigations incorporate longitudinal designs to assess causality or treatment effects. Additionally, inconsistencies in sample processing, metabolite identification, and normalization methods hinder reproducibility and cross‐study comparisons. Nevertheless, the collective evidence from metabolomics studies in RH demonstrates the feasibility of metabolite‐based biomarker discovery, the relevance of metabolic signatures for pathophysiological classification, and the potential utility in guiding precision therapy.

Moving forward, large, multicenter, prospective studies with standardized analytical pipelines and integration with clinical and multiomics data will be crucial. These efforts will validate existing findings, identify robust biomarkers, and facilitate clinical translation of metabolomics into RH management.

### 7.2. Replication Status of Candidate Metabolomic Markers

At present, no metabolite or metabolite panel can be considered a clinically validated biomarker specific for RH. Several candidate metabolites have been reported in RH‐specific or RH‐related studies, including citrate and oxaloacetate in urine metabolomics and treatment–response analyses, as well as ornithine, phenylalanine, uric acid, and indole derivatives in plasma metabolomics studies [12, 57]. Other pathways, including BCAAs, LysoPCs, sphingolipids, and acylcarnitines, are supported mainly by studies in essential hypertension, prehypertension, vascular aging, or drug‐response cohorts rather than confirmed RH cohorts [56, 59, 63]. Microbiota‐derived metabolites such as TMAO and SCFAs, as well as oxidative stress–related markers, provide mechanistic context but remain hypothesis‐generating for RH‐specific biomarker discovery [58, 65, 85, 86]. However, most findings remain exploratory because they have not been replicated in large, independent, ABPM‐confirmed, adherence‐verified RH cohorts [74, 76, 77, 80, 87, 88]. Therefore, these metabolites should be viewed as candidate markers requiring analytical validation, biological replication, and clinical qualification before translation into practice (Table 2).

**TABLE 2:** Replication status and interpretation of candidate metabolomic markers relevant to resistant hypertension.

Metabolite/pathway	Evidence in RH	Replication status	Suggested interpretation	References
Citrate, oxaloacetate, TCA cycle intermediates	Reported in RH‐related urine metabolomics and treatment–response studies	Limited; requires external validation	RH‐relevant exploratory marker	[57]
Ornithine, phenylalanine, uric acid, indole derivatives	Reported in RH plasma metabolomics	Limited; pilot‐level evidence	RH candidate marker	[12]
BCAAs	Mainly general hypertension, insulin resistance, obesity literature	Not RH‐specific	Extrapolated metabolic risk marker	[56, 59, 77, 80]
LysoPCs and sphingolipids	General hypertension and vascular aging studies	Not RH‐specific	Vascular/metabolic context marker	[68, 69, 72]
Acylcarnitines	General hypertension and drug‐response studies	Not RH‐specific	Mitochondrial/fatty acid oxidation marker	[63, 74, 76]
TMAO and SCFAs	Hypertension and microbiome studies	Direct RH evidence limited	Hypothesis‐generating microbiome marker	[65, 85, 86]
Allantoin, oxidative stress indicators	Hypertension/vascular oxidative stress literature	Not RH‐specific	Nonspecific oxidative stress marker	[58]

### 7.3. Directionality and Causality of Metabolomic Alterations

The directionality of metabolomic alterations in RH remains uncertain. Some metabolites may act as causal mediators that contribute to vascular dysfunction, sodium retention, oxidative stress, or inflammation. Others may represent consequences of sustained blood pressure elevation, renal impairment, target‐organ damage, or altered hemodynamics. In addition, many metabolic changes may reflect medication exposure or comorbid conditions such as CKD, diabetes, obesity, dyslipidemia, and obstructive sleep apnea. Cross‐sectional metabolomic studies cannot reliably distinguish these possibilities. Longitudinal designs, repeated sampling before and after treatment modification, Mendelian randomization, mechanistic cell or animal studies, and integration with pharmacometabolomics are needed to determine whether candidate metabolites are causal drivers, disease consequences, drug‐response markers, or comorbidity‐related signatures. These limitations highlight the need for multiomics integration and longitudinal validation to move beyond association‐based metabolite discovery toward mechanistic and clinically actionable biomarkers.

### 7.4. Pharmacometabolomic and Comorbidity‐Related Confounding

Pharmacometabolomic confounding is particularly important in RH because patients are exposed to multiple antihypertensive agents by definition, and medication exposure can substantially alter circulating metabolomic profiles [50, 63, 87–89]. Diuretics may influence uric acid, electrolyte balance, glucose metabolism, lipid metabolism, and volume‐related metabolites [63, 89, 90]. Mineralocorticoid receptor antagonists may alter steroid‐related pathways, aldosterone‐related biology, potassium handling, and spironolactone‐related drug metabolites [91–93]. Beta‐blockers can affect fatty acid oxidation, acylcarnitines, glucose–insulin metabolism, and triglyceride‐related pathways [63, 94, 95]. ACE inhibitors, ARBs, and calcium channel blockers may influence renal hemodynamics, vascular tone, inflammatory pathways, and lipid‐related pharmacometabolic features [89]. In addition, common concomitant medications in RH patients, including statins, antidiabetic drugs, aspirin, and NSAIDs, may affect lipid metabolism, amino acid metabolism, glucose metabolism, inflammatory mediators, prostaglandin‐related pathways, and renal metabolites [96–98]. Therefore, RH metabolomics studies should carefully document medication class, dose, duration, adherence, and recent treatment changes. Whenever possible, analyses should adjust for medication exposure, perform sensitivity analyses by drug class, and incorporate pharmacometabolomic designs to distinguish disease‐related signatures from drug‐induced metabolic changes. Although these studies provide important insights into metabolic perturbations associated with RH, the biological interpretation of these findings requires caution because the directionality, causality, and specificity of metabolite changes remain unresolved (Table 3).

**TABLE 3:** Potential pharmacometabolomic confounding by antihypertensive and concomitant medications in RH metabolomics studies.

Drug class	Potential metabolomic influence	Implication for RH metabolomics	References
Diuretics	Uric acid, electrolytes, glucose/lipid metabolism, volume‐related metabolites	May mimic or obscure RH‐associated metabolic signatures	[63, 89, 90]
Mineralocorticoid receptor antagonists	Steroid metabolism, aldosterone‐related pathways, potassium handling	May modify aldosterone‐dominant RH signatures	[91–93]
Beta‐blockers	Fatty acid oxidation, acylcarnitines, glucose/insulin‐related metabolites	May confound mitochondrial and lipid metabolism pathways	[63, 94, 95]
ACE inhibitors/ARBs	RAAS‐related metabolites, renal hemodynamics, inflammatory pathways	May alter vascular and renal metabolic profiles	[63, 89]
Calcium channel blockers	Vascular tone–related pathways, edema/volume‐related changes	May influence downstream vascular/metabolic readouts	[63, 89]
Statins, antidiabetic drugs, aspirin, NSAIDs	Lipids, amino acids, inflammatory mediators, renal metabolites	Important non‐antihypertensive confounders	[96–98]

## 8. Integration of Multiomics Approaches in RH Research

RH is a complex, multifactorial condition in which single‐layer omics analyses, such as metabolomics alone, often fall short in capturing the full biological context. The integration of multiple omics technologies, including genomics, transcriptomics, proteomics, epigenomics, and metabolomics, can provide a systems‐level understanding of RH, enabling comprehensive dissection of its pathophysiology and supporting the development of precision medicine approaches.

Genomic studies have identified genetic variants linked to blood pressure regulation, renal sodium handling, drug metabolism, and susceptibility to treatment resistance [99]. For instance, polymorphisms in genes encoding components of the RAAS or sodium transporters have been associated with RH. These findings offer insights into inherited predispositions and may eventually inform genotype‐guided therapy [100].

Transcriptomics adds another layer of insight by characterizing gene expression changes in response to hypertensive stimuli [101]. Altered expression of proinflammatory cytokines, oxidative stress markers, and vascular remodeling genes has been reported in RH patients. These changes reflect active disease processes and may help identify transcriptional biomarkers or therapeutic targets.

Proteomics complements transcriptomic data by profiling changes in protein abundance, post‐translational modifications, and protein–protein interaction networks. In RH, proteomic studies have highlighted alterations in inflammatory mediators, extracellular matrix components, and enzymes involved in redox regulation. Such information provides mechanistic insight into vascular dysfunction and drug resistance [102].

Metabolomics serves as a convergence point of upstream genetic and proteomic alterations and downstream environmental influences. Integrating metabolomics with other omics layers enables the construction of integrative models that reveal key regulatory nodes and interconnected pathways [103].

A particularly exciting area of RH research involves the gut microbiome. Metagenomic sequencing of the gut microbiota has identified specific bacterial taxa associated with blood pressure regulation [104]. When combined with metabolomics, these data reveal host–microbe interactions that influence inflammation, RAAS activity, and vascular tone through metabolites such as SCFAs, trimethylamine, and TMAO [86].

Epigenomics also plays a role, particularly in the context of environmental exposures (e.g., diet, stress, and medication) that may modify gene expression without altering DNA sequence. DNA methylation and histone modification patterns may influence hypertension susceptibility and contribute to interindividual variability in treatment response [105].

To manage and interpret the high‐dimensional data generated by multiomics studies, advanced computational tools, including network analysis, machine learning, and artificial intelligence, are essential. These tools can be used to identify RH subtypes, predict therapeutic outcomes, and prioritize candidate biomarkers for validation [106].

The integration of multiomics data may provide a useful framework for future RH research and clinical translation. By illuminating the molecular complexity of RH, this approach enables the development of more accurate diagnostics, individualized therapies, and predictive models that move beyond conventional clinical algorithms.

## 9. Clinical Translation and Precision Medicine in RH

### 9.1. Proposed Clinical Workflow for Metabolomics‐Guided RH Management

**FIGURE 2:**
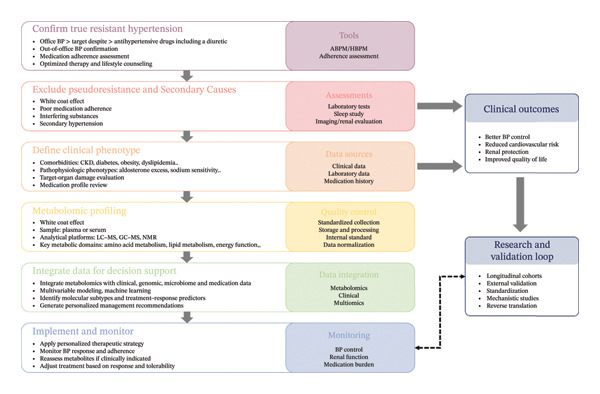
Proposed clinical workflow for metabolomics‐guided management of resistant hypertension. The workflow positions metabolomics as an adjunctive tool after confirmation of true resistant hypertension using standardized blood pressure assessment, ABPM or HBPM, medication adherence evaluation, and optimized therapy. Metabolomic profiling is integrated with clinical phenotyping, medication history, and multiomics data to support risk stratification, treatment–response prediction, and longitudinal monitoring.

Metabolomics should be positioned as an adjunctive tool within a stepwise RH care pathway rather than as a standalone diagnostic test. A proposed workflow includes (1) confirmation of true RH using standardized office blood pressure, ABPM, or HBPM, adherence assessment, and optimized therapy; (2) clinical phenotyping based on CKD, diabetes, obesity, obstructive sleep apnea, aldosterone excess, sodium sensitivity, and target‐organ damage; (3) metabolomic profiling to identify pathway‐level signatures related to volume expansion, RAAS activation, mitochondrial dysfunction, oxidative stress, inflammation, or gut microbiota–derived metabolites; (4) integration of metabolomic data with clinical variables and other omics layers to support risk stratification and treatment–response prediction; and (5) longitudinal monitoring to assess biochemical response after medication adjustment, lifestyle intervention, or device‐based therapy (Figure 2).

### 9.2. Clinical Reverse Translation

Clinical reverse translation refers to the process of utilizing patient‐derived clinical and molecular data to generate hypotheses that can be tested in preclinical models, thereby closing the loop between bench and bedside [107]. This approach is particularly valuable in diseases like RH, which are heterogeneous and influenced by both genetic and environmental factors [4].

Metabolomics serves as a powerful tool in clinical reverse translation by offering real‐time snapshots of physiological and pathological states [108]. Unlike static genetic data, metabolomic profiles dynamically reflect the cumulative effects of gene–environment interactions, disease activity, and pharmacologic interventions. In RH, this allows researchers to identify metabolic signatures associated with treatment resistance, target‐organ damage, or cardiovascular risk and subsequently investigate their mechanistic roles using experimental models [109].

For instance, an elevated profile of BCAAs, oxidative stress markers, or gut microbiota–derived metabolites such as TMAO in RH patients could prompt mechanistic studies examining their role in endothelial dysfunction, vascular tone regulation, or inflammation [87]. These patient‐informed hypotheses can be tested in animal or cellular models, enabling causality to be established and new drug targets to be validated.

Furthermore, metabolomics can stratify RH patients into clinically meaningful subgroups with differing pathophysiological profiles, such as aldosterone‐dominant RH versus insulin resistance–driven RH [110]. This stratification enhances the design of tailored interventions and supports personalized prescribing. For example, a subset of RH patients with metabolomic evidence of aldosterone excess may benefit from mineralocorticoid receptor antagonists, while others with markers of mitochondrial dysfunction might respond better to agents targeting oxidative phosphorylation [111].

Metabolomics also supports dynamic treatment monitoring by capturing early biochemical changes in response to therapy, allowing for rapid treatment adjustments. This can improve therapeutic efficacy, reduce unnecessary drug exposure, and optimize long‐term outcomes [112].

In summary, metabolomics enables a bidirectional translational loop between clinical observations and preclinical experimentation. It helps elucidate disease mechanisms, discover new therapeutic targets, and guide precision medicine approaches in RH. The integration of metabolomics into clinical reverse translation represents a critical step toward data‐driven, individualized care in RH.

### 9.3. Validation Standards for Clinical Implementation

Before clinical implementation, RH metabolomic biomarkers must satisfy several validation standards, including analytical validity, clinical validity, predictive performance, incremental value, clinical utility, cost‐effectiveness, and generalizability (Table 4) [112–115]. Analytical validity requires reproducible assays, standardized biospecimen collection, robust metabolite identification, quality control procedures, batch correction, and interlaboratory reproducibility [113–115]. Clinical validity requires external validation in independent cohorts with confirmed true RH, preferably using ABPM or HBPM, medication adherence assessment, optimized treatment documentation, and exclusion of pseudoresistance [87, 88, 116]. Predictive models should demonstrate adequate discrimination, calibration, sensitivity, specificity, decision‐curve performance, and incremental value beyond standard clinical variables [117–119]. Finally, clinical utility must be shown by demonstrating that metabolomics‐guided decisions improve blood pressure control, reduce medication burden, predict treatment response, improve cardiovascular or renal outcomes, and provide cost‐effective value compared with usual care [119, 120].

**TABLE 4:** Validation standards for clinical implementation.

Validation domain	Minimum requirement	References
Analytical validity	Reproducible assay, standardized sample collection, batch correction, interlaboratory reliability	[113–115]
Clinical validity	External validation in ABPM‐confirmed, adherence‐verified RH cohorts	[87, 88, 116]
Predictive performance	AUC, sensitivity, specificity, calibration, decision‐curve analysis	[116–118]
Incremental value	Improvement beyond age, sex, BP, CKD, diabetes, obesity, medications, and standard laboratory variables	[117–119]
Clinical utility	Evidence that metabolomics‐guided decisions improve BP control, target‐organ damage, cardiovascular outcomes, or medication burden	[112, 119]
Cost‐effectiveness	Acceptable cost, turnaround time, scalability, simplified targeted assay	[120]
Generalizability	Validation across sex, ethnicity, CKD, diabetes, obesity, dietary patterns, and medication backgrounds	[113, 117, 118]

## 10. Future Directions and Conclusion

RH continues to pose a significant challenge in cardiovascular medicine due to its complex etiology, high prevalence, and poor prognosis. Despite advancements in pharmacologic therapy and the development of interventional strategies, current management approaches often fail to achieve optimal blood pressure control in a large subset of patients. The biological heterogeneity of RH underscores the necessity for precision medicine strategies that are informed by molecular profiling and systems‐level understanding [121].

Metabolomics has emerged as a particularly promising tool in this context. It provides an integrative view of metabolic alterations that reflect genetic background, environmental exposures, comorbid conditions, and medication responses. By characterizing the biochemical landscape of RH, metabolomics can enhance disease classification, facilitate early diagnosis, predict therapeutic response, and uncover novel targets for treatment [122, 123].

Looking ahead, the field should prioritize large‐scale, longitudinal studies that apply standardized metabolomic protocols across diverse populations. Harmonization of sample collection, analytical platforms, quality control procedures, and data normalization will be essential to improve reproducibility and facilitate cross‐cohort comparisons. These studies should be embedded within clinical cohorts and incorporate detailed phenotyping, including ABPM or HBPM, adherence assessment, comorbidity profiles, and medication histories [88, 113].

Furthermore, integration of metabolomics with other omics layers, such as genomics, transcriptomics, proteomics, and microbiomics, will be essential for capturing the full complexity of RH pathophysiology. Multiomics approaches combined with machine learning and artificial intelligence can uncover novel disease subtypes, identify predictive biomarkers, and guide the development of individualized therapeutic algorithms [124].

Finally, translational research must focus on making metabolomics more clinically accessible by developing simplified targeted assays, user‐friendly analytic pipelines, validated prediction models, evidence‐based clinical decision‐support tools, and cost‐effective implementation strategies [114]. Interdisciplinary collaboration among clinicians, biostatisticians, bioinformaticians, and laboratory scientists will be vital in overcoming current challenges.

In conclusion, metabolomics provides a promising systems‐level approach for understanding the biological heterogeneity of RH and identifying candidate biomarkers linked to vascular, renal, inflammatory, neurohormonal, metabolic, and microbiome‐related pathways. However, current evidence is largely exploratory, and few findings have been replicated in independent cohorts with rigorously confirmed true RH. Future studies should incorporate ABPM or HBPM, medication adherence assessment, optimized treatment documentation, longitudinal sampling, drug‐exposure adjustment, and external validation. Metabolomics is therefore best viewed at present as a research and phenotyping tool rather than a clinically established diagnostic test. With rigorous validation and integration into clinical workflows, metabolomics may ultimately contribute to precision medicine strategies for RH.

## Author Contributions

Yu Ra Lee: data curation, visualization, writing–original draft. Miri Park: writing–original draft. Yoon Shin Cho: investigation, writing–review and editing. Ho‐Young Park: conceptualization, writing–review and editing.

## Funding

This study was supported by the Korea National Institute of Health, 2024‐ER0913‐00.

## Conflicts of Interest

The authors declare no conflicts of interest.

## Data Availability

Data sharing is not applicable to this article, as no datasets were generated or analyzed during the current study.
